# Incidence of seroma in sublay versus onlay mesh repair of incisional hernia

**DOI:** 10.1016/j.amsu.2020.12.029

**Published:** 2020-12-30

**Authors:** Rashid Ibrahim, Sabry Abounozha, Adel Kheder, Talal Alshahri

**Affiliations:** a Department of General Surgery, University Hospitals Plymouth NHS Trust, Plymouth, UK; b Department of General Surgery, Northumbria Healthcare NHS Foundation Trust, Northumbria, UK; c Department of General Surgery, University Hospital Southampton NHS Foundation Trust, Southampton, UK; d Department of General Surgery, Imam Abdulrahman Alfaisal Hospital, Riyadh, Saudi Arabia

**Keywords:** Incisional hernia, Onlay, Seroma, Sublay

## Abstract

A best evidence topic has been constructed using a described protocol. The three-part question addressed was: In patient undergoing open mesh repair of incisional hernia, is there any difference in the rate of seroma between Sublay and Onlay technique? The best evidence showed that Sublay repair has a lower seroma rate in comparison to onlay repair.

## Highlights

• Aim: Comparison of seroma rate among patients who underwent Onlay vs Sublay mesh repair of incisional hernia.
• Result: Six trials, including three randomized controlled.
• Conclusion: The best evidence showed that sublay mesh repair has lower rate of seroma compared to only repair.

## 1 Introduction

This BET was designed using a framework outlined by the International Journal of Surgery [1]. This format was used because a preliminary literature search suggested that the available evidence is of insufficient quality to perform a meaningful meta-analysis. A BET provides evidence-based answers to common clinical questions, using a systematic approach of reviewing the literature. (see Table 1)

**Table 1:** Summary of the articles.

Author, date of publication, journal and country	Study type and level of evidence	Patient group	Outcomes Follow up	Key results		Additional comments
Sevinç et al. [7] Turk J Surg Turkey	Randomized controlled trial level II	Total of 100 with incisional hernia **Group 1**: 50 sublay **Group2**: 50 onlay median follow-up was 37.1 (26.6–46.5) months	Primary endpoint: Incidence of seroma	(ER) rate was: Group1 = 1 (2%) Group2 = 7 (14%) P = 0.027 Difference is statistically significant		-Single centre, -Small sample size, -Short period of follow up
Venclauskas et al. [8] Hernia Lithuania	Randomized controlled trial level II	Total 107 patients underwent mesh repair Group1: 57 onlay Group2: 50 sublay median follow-up 12 months	Primary endpoint: Incidence of seroma	Group1: 26 (45%) Group2: 12 (24%) P = 0.02 Difference is statistically significant		Single centre, -Small sample size, -Short period of follow up
Ahmed and Manzoor. [9] J Coll Physicians Surg Pak, Pakistan	Multicenter, Randomized, Controlled Trial levelII	Total 65 patients underwent mesh repair Group1: 33 onlay Group2: 32 sublay median follow-up Six months.	Primary endpoint: Incidence of seroma	Group1: 13 (20%) Group2: 3 (4.61%) (P = 0.005). Difference is statistically significant		-Small sample size, -Short period of follow up
Kumar et al. [5] Indian J Surg India	Prospective study level III	Total 63 patients Randomized into: **Group 1**: 45 onlay **Group 2**: 18 sublay follow-up 5 years	Primary endpoint: Incidence of seroma	**Group 1** = 11 (24.44%) **Group 2 =** 5 (27.77%) Difference is not statistically significant	-Single centre, -Small sample size -No randomization - sample size is not equal between 2 groups	
John J. Gleysteen. [6], Arch Surg, UK	Retrospective study level III	A total of 125 patients: **Group 1: 75 onlay Group 2: 50 sublay** Group 1: 75 onlay Group 2: 50 sublay Follow-up periods averaged 64 months	Primary endpoint: Incidence of seroma	**Group1** = 16 (21.3%) **Group2** = 12 (24.0%) (P = 0.58) Difference is not statistically significant	-Single centre, -small sample size, -Retrospective –	
Reilingh et al. [10] Hernia Netherlands	Retrospective cohort study, level III	36 patients underwent Mesh repair of incisional hernia Group 1: 13 onlay Group 2: 23 sublay	Primary endpoint: Incidence of seroma	**Group 1** = 9 (69%) **Group 2** = 0 (0.0%) (P < 0.05) Statically significant	--Single centre, -small sample size, -Retrospective	

## 2 Clinical scenario

You are consenting a 45 year old female with recurrent incisional hernia for open mesh repair the pervious operation was complicated with seroma, the patient is wondering which technique provide a lower seroma rate onlay or sublay mesh repair ?

## 3 Three-part question

• [In patient undergoing open mesh repair of incisional hernia]
• [is there any difference in the rate of seroma]
• [between Sublay and Onlay technique]?

## 4 Search strategy

### 4.1 Embase 1974 to October 2020 using the OVID interface

[Incisional hernia] AND [mesh] AND [repair OR repairs] AND [onlay] AND [sublay] AND [seroama]
B. Medline using the PubMed interface:
[Incisional hernia] AND [mesh] AND [repair OR repairs] AND [onlay] AND [sublay] AND [ seroma]
• **Inclusion criteria**: all original articles in children or adults which compare the incidence of seroma among the Onlay and Sublay mesh repair of incisional hernia
• **Exclusion criteria**: case reports, letter to the editor, conference abstract, systematic review, articles not in English.

## 5 Search outcome

**Fig. 1:**
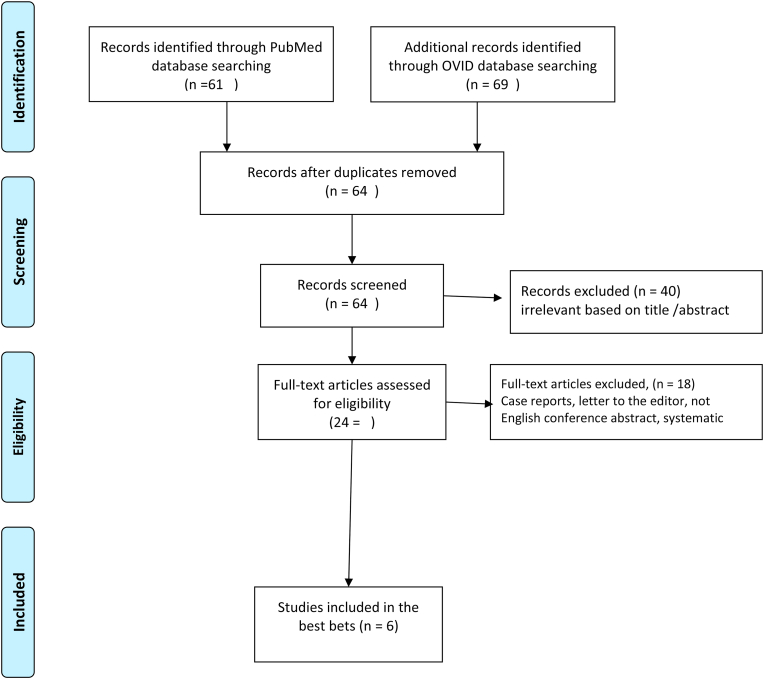
PRISMA flow diagram

A total of 64 articles were identified after the removal of duplicates. Of these 40 were excluded because they are irrelevant on the basis of title and abstract. After full-text assessment of 24 articles another 16 articles were excluded because they met one of the exclusion criteria above. A total of 6 articles (2 randomized controlled trials, one prospective and 3 retrospective studies) were identified to provide the best evidence to answer the question see (Fig. 1).

## 6 Result

Total of six articles were included in our review; three randomized control trials (RCTs), one prospective study and two retrospective studies. All the studies were conducted in a single centres except on was multicentre. Four out of six articles showed a statistically significant lower seroma rate among those patients who had Sublay mesh repair compared to Onlay technique.

## 7. discussion

7

Incisional hernia remains one of the most common postoperative complications following abdominal surgery, with incidences ranging from 11% to 20% [2]. Seroma formation is a common complication after incisional hernia repair, especially with the use of mesh. The incidence of seroma is ranging from 30 to 50% after open mesh repair. The exact pathophysiology of seroma formation is unknown. Although it is postulated that the presence of mesh will act as a foreign body that induce local inflammation and fluid accumulation [3]. In addition, the larger space created by dissection of the hernia sac and the fascial clearances which typically occur in onlay mesh technique will increase the risk of local wound complications such as hematoma, seroma, and infection [4].

In this article, we have reviewed the best studies which compared the two most common modalities of open mesh repair of incisional hernia (Onlay and Sublay) in order to evaluate which techniques has the lower incidence of seroma formation.

Two studies in our review showed no statistically significant difference in seroma rate between onlay and sublay mesh repair these studies were conducted by Kumar et al. [5] and Gleysteen [6]. However both studies are of low quality. Evidence not randomized control trials, and they have small sample size.

In contrast, the rest of the four studies showed statistically significant lower incidence of seroma among the sublay group of patients in comparison to the onlay group these include: three randomized control trials and one retrospective study [[7], [8], [9], [10]].

### 7.1 Clinical bottom line

According to the above articles, the best evidence showed a statistically significant low rate of seroma among sublay mesh repair of incisional hernia in comparison to Only repair.

### 7.2 Limitation of this review

1. Small sample size in most articles: the largest sample size included was 125 patents which is relatively small number for a common problem such as incisional hernia
2. All studies except were single centre studies: again this might be the reason behind the small sample size.
3. In all articles there is nothing mentioned about the exact definition or classification of seroma and how it was diagnosed or treated.

* in order to overcome this limitations; the authors do recommend a future large size multicentre well designed randomized controlled trail in order to draw a better conclusion about which techniques is associated with lower seroma rate.

## Ethical approval

Not applicable.

## Sources of funding

None.

## CRediT authorship contribution statement

**Rashid Ibrahim:** conducted the literature search and wrote the paper. **Sabry Abounozha:** assisted in the literature search and Writing of paper. **Adel Kheder:** assisted in writing of paper. **Talal Alshahri:** assisted in the literature search, editing of writing.

## Declaration of competing interest

None.
